# Identification and Characterization of a Novel Chromosomal Aminoglycoside 2′-*N*-Acetyltransferase, AAC(2′)-If, From an Isolate of a Novel *Providencia* Species, *Providencia wenzhouensis* R33

**DOI:** 10.3389/fmicb.2021.711037

**Published:** 2021-11-19

**Authors:** Kexin Zhou, Jialei Liang, Xu Dong, Peiyao Zhang, Chunlin Feng, Weina Shi, Mengdi Gao, Qiaoling Li, Xueya Zhang, Junwan Lu, Xi Lin, Kewei Li, Hailin Zhang, Mei Zhu, Qiyu Bao

**Affiliations:** ^1^The Second Affiliated Hospital and Yuying Children’s Hospital, Wenzhou Medical University, Wenzhou, China; ^2^Key Laboratory of Medical Genetics of Zhejiang Province, Key Laboratory of Laboratory Medicine, Ministry of Education, School of Laboratory Medicine and Life Sciences, Wenzhou Medical University, Wenzhou, China; ^3^Institute of Biomedical Informatics, School of Laboratory Medicine and Life Sciences, Wenzhou Medical University, Wenzhou, China; ^4^Department of Clinical Laboratory, Zhejiang Hospital, Hangzhou, China

**Keywords:** *Providencia*, aminoglycoside, AAC(2cpsdummy′), resistance, kinetic analysis

## Abstract

Multidrug-resistant bacteria from different sources have been steadily emerging, and an increasing number of resistance mechanisms are being uncovered. In this work, we characterized a novel resistance gene named *aac(2′)-If* from an isolate of a novel *Providencia* species, *Providencia wenzhouensis* R33 (CCTCC AB 2021339). Susceptibility testing and enzyme kinetic parameter analysis were conducted to determine the function of the aminoglycoside 2′-*N*-acetyltransferase. Whole-genome sequencing and comparative genomic analysis were performed to elucidate the molecular characteristics of the genome and the genetic context of the resistance gene-related sequences. Among the functionally characterized resistance genes, AAC(2′)-If shares the highest amino acid sequence identity of 70.79% with AAC(2′)-Ia. AAC(2′)-If confers resistance to several aminoglycoside antibiotics, showing the highest resistance activity against ribostamycin and neomycin. The recombinant strain harboring *aac(2′)-If* (pUCP20-*aac(2′)-If*/DH5α) showed 256- and 128-fold increases in the minimum inhibitory concentration (MIC) levels to ribostamycin and neomycin, respectively, compared with those of the control strains (DH5α and pUCP20/DH5α). The results of the kinetic analysis of AAC(2′)-If were consistent with the MIC results of the cloned *aac(2′)-If* with the highest catalytic efficiency for ribostamycin (*k_*cat*_/K_*m*_* ratio = [3.72 ± 0.52] × 10^4^ M^–1^^⋅^s^–1^). Whole-genome sequencing demonstrated that the *aac(2′)-If* gene was located on the chromosome with a relatively unique genetic environment. Identification of a novel aminoglycoside resistance gene in a strain of a novel *Providencia* species will help us find ways to elucidate the complexity of resistance mechanisms in the microbial population.

## Introduction

Aminoglycoside antibiotics disturb translation and promote mistranslation of proteins, resulting in altering the integrity of bacterial cell membranes (Aguirre Rivera et al., 2021). They have a broad antimicrobial spectrum, and they often work in synergy with other antibiotics, making them valuable anti-infective agents (van Hoek et al., 2011). With the widely growing use of antibiotics in human clinical or agricultural practices, antibiotic resistance is a growing problem associated with the use of all classes of anti-infective agents. Aminoglycoside resistance has many different forms. The major aminoglycoside resistance mechanism encountered in clinical isolates of gram-negative and gram-positive bacteria is most frequently associated with the expression of modifying enzymes (Wright, 1999; Vakulenko and Mobashery, 2003). To date, more than 100 aminoglycoside-modifying enzymes (AMEs) have been described and are broadly categorized into three groups based on the type of modification (Ramirez and Tolmasky, 2010). These three families of AMEs include acetyltransferases (AACs), nucleotidyltransferases or adenyltransferases (ANTs), and phosphotransferases (APHs). These classes are further divided into subtypes based on different region specificities of the enzymes for aminoglycoside modifications (Shaw et al., 1993; Ramirez and Tolmasky, 2010). The subclasses include four AACs, namely, AAC(1), AAC(2′), AAC(3), and AAC(6′); five ANTs, namely, ANT(2′′), ANT(3′′), ANT(4′), ANT(6), and ANT(9); and seven APHs, namely, APH(2′′), APH(3′), APH(3′′), APH(4), APH(6), APH(7′′), and APH(9) (Haas and Dowding, 1975; Shaw et al., 1993).

Acetyltransferases belong to the ubiquitous GCN5-related *N*-acetyltransferase (GNAT) superfamily of proteins, which includes approximately 10,000 proteins (Vetting et al., 2005). AACs are classified according to the sites of acetylation of the deoxystreptamine core of aminoglycoside antibiotics (Shaw et al., 1993). Most of the genes encoding these enzymes are plasmid borne, but an exception to this general rule is the 2′-*N*-acetyltransferase [AAC(2′)-Ia] from *Providencia stuartii*, which is chromosomally encoded (Rather et al., 1993; Payie et al., 1995). Additionally, AAC(2′)-Ia has been identified as the most prevalent chromosomally encoded AME among *P. stuartii* strains (Clarke et al., 1996; Franklin and Clarke, 2001), while AAC(2′) enzymes (AAC(2′)-Ib, AAC(2′)-Ic, AAC(2′)-Id, and AAC(2′)-Ie) have been found in different species of the genus *Mycobacterium* (Rather et al., 1993; Ainsa et al., 1996, 1997; Hegde et al., 2001; Adams et al., 2008). In the wild-type *P. stuartii*, the *aac(2′)-Ia* gene is normally expressed at low levels, and it is regulated in part by a small transcriptional activator and at least two other trans-acting negative regulators (Macinga et al., 1998; Ding et al., 2001).

*Providencia* species belong to the order Enterobacterales and family Morganellaceae, which is a family of gram-negative opportunistic human pathogens. *Providencia* species closely resemble *Proteus* and *Morganella* species and are quite ubiquitous in the environment (Sagar et al., 2017). Currently, 13 *Providencia* species are recognized: *P. stuartii*, *P. rettgeri*, *P. rustigianii*, *P. heimbachae*, *P. alcalifaciens*, *P. burhodogranariea*, *P. sneebia*, *P. vermicola*, *P. huaxiensis*, *P. thailandensis*, *P. entomophila*, *P. friedericiana*, and *Candidatus P. siddallii*^1^. *P. rettgeri*, one of the most common pathogens of *Providencia* spp., has been isolated from samples collected from patients with various infectious diseases, such as nephrocutaneous fistula (Lee and Hong, 2011), urinary tract infections (Barrios et al., 2013), and soft tissue infections (Carvalho-Assef et al., 2013).

In this work, we report the identification and characterization of a novel aminoglycoside 2′*-N-*acetyltransferase designated AAC(2′)-If encoded in the chromosome of a *Providencia wenzhouensis* strain isolated from a rabbit. In addition, based on sequence analysis, the genetic environment of the *aac(2′)-If* gene and its genetic relationship with other AACs were also analyzed.

## Materials and Methods

### Bacterial Strains and Plasmids

To investigate the antimicrobial resistance of large intestinal bacteria in animals, we collected anal feces samples from different animals. *P. wenzhouensis* R33 was isolated from an anal swab of a New Zealand White rabbit in an animal farm in Wenzhou, southeastern China. The anal swab was collected as follows: a sterile cotton swab was inserted into the anus of rabbit for 3–5 cm, gently rotated, and then immediately put it into a sterilized screw-capped specimen collection tube containing normal saline solution (0.9% sodium chloride). The isolate was randomly isolated by the streak plate method with a normal Luria-Bertani agar plate, initially identified using the Vitek-60 microorganism autoanalysis system (BioMerieux Corporate, Craponne, France) and verified by analysis of the 16S rRNA gene sequences. The result was finally confirmed by determining the average nucleotide identity (ANI) using FastANI and *in silico* DNA–DNA hybridization (*is*DDH) (Jain et al., 2018). The strains and plasmids used in this work are listed in Table 1.

**TABLE 1:** Bacteria and plasmids used in this work.

**Strain or plasmid**	**Relevant characteristic(s)**	**Reference or source**
Strain		
R33	The wild-type strain of *Providencia wenzhouensis* R33	This study
DH5α	*E. coli* DH5α was used as a host for the cloning of the *aac(2′)-If* gene	Our laboratory collection
RosettagamiB (DE3)	*E. coli* RosettagamiB was used as a host for expression of AAC(2′)-If	Our laboratory collection
ATCC 25922	*E. coli* ATCC 25922 was used as a quality control for antimicrobial susceptibility testing	Our laboratory collection
pUCP20-*aac(2′)-If*/DH5α	DH5α carrying the recombinant plasmid pUCP20-*aac(2′)-If*	This study
pCold I-*aac(2′)-I f/*RosettagamiB	RosettagamiB carrying the recombinant plasmid pCold I-*aac(2′)-If*	This study
Plasmid		
pUCP20/DH5α	Cloning vector for the PCR products of the *aac(2′)-If* gene with its upstream promoter region, ampicillin resistance	Our laboratory collection
pCold I/RosettagamiB	Expression vector for the PCR products of the ORF of the *aac(2′)-If* gene, ampicillin resistance	Our laboratory collection

### Antimicrobial Susceptibility Testing

The minimum inhibitory concentrations (MICs) were determined using the agar dilution method with Mueller-Hinton (MH) agar plates following the guidelines of the Clinical and Laboratory Standards Institute (CLSI). Susceptibility patterns were interpreted according to the CLSI breakpoint criteria (CLSI, 2019) and the guidelines of the European Committee on Antimicrobial Susceptibility Testing (The European Committee on Antimicrobial Susceptibility Testing, 2019) for Enterobacteriaceae. *Escherichia coli* ATCC 25922 was used as a reference strain for quality control. The MIC was determined in triplicate on Mueller-Hinton (MH) agar plates with two-fold serial dilutions of the antibiotics. The plates were incubated at 37°C for 16–20 h before analyzing the results.

### Whole-Genome Sequencing and Sequence Analysis

Genomic DNA of *P. wenzhouensis* R33 was extracted from bacterial culture by an AxyPrep Bacterial Genomic DNA Miniprep Kit (Axygen Scientific, Union City, CA, United States). DNA sequencing was performed by using the Illumina HiSeq-2500 and PacBio RS II platforms by the Shanghai Personal Biotechnology Co., Ltd. (Shanghai, China). The Illumina short reads and PacBio long reads were initially assembled by Canu v2.1 (Koren et al., 2017) and SPAdes v3.14.1 (Bankevich et al., 2012), respectively. Pilon v1.23 (Walker et al., 2014) was employed for further correction to improve assembly quality by mapping short reads aligned to the draft of the whole-genome assembly. Potential open reading frames (ORFs) of the assembled genome were predicted using Prokka v1.14.6 (Seemann, 2014), and functional annotation of these proteins was performed by BLAST analysis with an e-value threshold of 1e-5 against the nonredundant protein sequence database of US National Center for Biotechnology Information (NCBI) and the UniProt/Swiss-Prot database. Antimicrobial resistance genes were annotated using ResFinder (Zankari et al., 2012) and Resistance Gene Identifier (RGI) v4.0.3 of the Comprehensive Antibiotic Resistance Database (CARD) (McArthur et al., 2013). Mobile genetic elements (MGEs) were detected using ISFinder (Siguier et al., 2006) and INTEGRALL (Moura et al., 2009). ANI was calculated using FastANI v1.31 (Jain et al., 2018). CGView Server (Petkau et al., 2010) was used to visualize the basic genomic features of chromosomes and perform comparative genomic analysis. The promoter region of *aac(2′)-If* was predicted by BPROM (2016)^2^. The molecular weight and pI value of AAC(2′)-If were predicted using Expasy ProtParam Tool (2021)^3^. Multiple alignment of the amino acid sequences of and neighbor-joining phylogenetic tree construction for AAC(2′)-If and other AACs were performed using MAFFT v7.475 (Katoh and Standley, 2013) and MEGAX (Kumar et al., 2018), respectively. The sequence retrieval, statistical analysis, and other bioinformatics tools used in this study were written using Python (2021)^4^ and Biopython (Cock et al., 2009).

### Cloning of the *aac(2′)-If* Gene

The coding sequence of *aac(2′)-If*, along with its upstream promoter region, was amplified by PCR primers with a pair of flanking restriction endonuclease adaptors for *Bam*HI and *Hin*dIII (Takara Bio, Inc., Dalian, China) introduced at the 5′ and 3′ ends, respectively (Supplementary Table S1). The PCR product was digested with *Bam*HI and *Hin*dIII, and then ligated into the pUCP20 vector with a T4 DNA ligase cloning kit (Takara Bio, Inc., Dalian, China). The recombinant plasmid pUCP20-pro-*aac(2′)-If* was transformed into competent *E. coli* DH5α cells by the calcium chloride method (Chan et al., 2013), and the transformant was selected on Luria-Bertani agar plates supplemented with 100 μg/mL ampicillin. The cloned insert sequence (*aac(2′)-If* with its upstream promoter region) in the recombinant plasmid of the transformant was further confirmed by both restriction enzyme digestion and Sanger sequencing (Shanghai Sunny Biotechnology Co., Ltd., Shanghai, China).

### Expression and Purification of Recombinant AAC(2′)-If

The ORF of the *aac(2′)-If* gene was amplified with the orf-*aac(2′)-If* primers listed in Supplementary Table S1 and cloned into the pCold I cold shock expression vector (Qing et al., 2004). The resultant plasmid pCold I-*aac(2′)-If* was introduced into *E. coli* RosettagamiB (DE3) competent cells, and transformants (pCold I-orf-*aac(2′)-If*/RosettagamiB) were selected on LB agar plates containing 100 μg/mL ampicillin.

The overnight culture of the recombinant strain (pCold I-orf-*aac(2′)-If*/RosettagamiB) was cultured in LB broth containing 100 μg/mL ampicillin at 37°C. When the OD at 600 nm reached 0.6, isopropyl D-thiogalactopyranoside (IPTG) was added at a concentration of 1 mM to induce the expression of AAC(2′)-If, and cell cultivation was continued for 18 h at 16°C. The cells were collected, resuspended in phosphate buffered saline (pH = 7.4), and disrupted with a French pressure cell. The recombinant protein was purified using BeyoGold His-tag Purification Resin and subsequently eluted by the nondenatured eluent (50 mM NaH_2_PO_4_, 300 mM NaCl, 50 mM imidazole) of the His-tag Protein Purification Kit (Beyotime, Shanghai, China) according to the manufacturer’s instructions. The purity of the protein was confirmed using sodium dodecyl sulfate polyacrylamide gel electrophoresis (SDS-PAGE) and subsequent staining with Coomassie Brilliant Blue. The protein concentration was determined spectrophotometrically using a BCA protein assay kit (Thermo Fisher Scientific, Rockford, IL, United States).

### RT Q-PCR Assays

To analyze the expression of *aac(2′)-If*, total RNA was extracted from the bacteria cultured in LB broth grown to OD_600_ = 0.5 and then treated with or without the addition of 1/4 MIC ribostamycin (32 μg/mL), neomycin (1 μg/mL), and gentamicin (0.25 μg/mL) for 0.5, 1, 2, 4, and 24 h, respectively. Then total RNA was isolated using TRIzol reagent (Sigma, Shanghai, China) according to the manufacturer’s protocol and quantified by an Implen NanoPhotometer (Implen GmbH, Munich, Germany). DNA-free RNA was confirmed by PCR amplification of the *Providencia* 16S rRNA gene. cDNA was synthesized using the PrimeScript RT-PCR Kit (Takara Bio, Inc., Dalian, China). RT Q-PCR was performed using the corresponding cDNA from each sample. The primers used for RT Q-PCR are listed in Supplementary Table S2. RT Q-PCR was performed in a CFX96^TM^ Touch Real-Time PCR Detection System (Bio-Rad Laboratories, Hercules, CA, United States) by monitoring the increase in fluorescence in real time using SYBR qPCR Master Mix (Vazyme Biotech, Nanjing, China). Relative quantification was performed using the CT method (Livak and Schmittgen, 2001) with the housekeeping genes 16S rRNA as references. Comparisons of expression levels with or without antibiotics treatment were analyzed using one-way ANOVA (LSD test). *P* ≤ 0.05 was considered significant.

### Kinetic Studies of AAC(2′)-If

The kinetic parameters for AAC(2′)-If activity were measured spectrophotometrically by following the production of coenzyme A (CoASH), which was produced upon the transfer of the acetyl moiety to the aminoglycoside. The thiol group of CoASH reacts with 5,5′-dithiobis(2-nitrobenzoic acid) (DTNB), which is replaced by 4,4′-dithiodipyridine, and monitoring was performed at 412 nm to measure an increase in the absorbance of the formed product, pyridine-4-thiolate (TNB), as previously described (Hegde et al., 2001; Galimand et al., 2015). Kinetic assays were performed in a 200 μl reaction mixture containing 25 mM 2-(*N*-morpholino)ethanesulfonic acid (MES; pH 6.0), 1 mM EDTA, 80 μM acetyl-CoA, 2 mM DTNB, and variable concentrations of aminoglycosides (5–150 μM) (Franklin and Clarke, 2001), and the reactions, initiated by the addition of 2 μg purified enzyme (final concentration), were monitored using a Synergy Neo2 Multi-Mode Microplate Reader (Biotek, VT, United States) at room temperature for 10 min. The steady-state kinetic parameters (*k*_*cat*_ and *K*_*m*_) were determined by nonlinear regression of the initial reaction rates with the Michaelis–Menten equation using GraphPad Prism 9 (GraphPad Software, San Jose, CA, United States).

### Nucleotide Sequence Accession Numbers

The *aac(2′)-If* sequence has been assigned GenBank accession number MW984427. The complete genome sequence of *P. wenzhouensis* R33 presented in this study has been deposited in GenBank under accession numbers CP072453 and CP072454 for chromosome and plasmid pR33-1 of *P. wenzhouensis* R33, respectively.

## Results

### Identification and Characteristics of the AAC(2′)-If-Producing Isolate of *Providencia wenzhouensis* R33

A 16S ribosomal RNA gene homology analysis showed that the 16S rRNA gene of strain R33 shared the highest similarity with that of *P. vermicola* strain OP1 (NR_042415.1), with 98.01% identity and 99.00% coverage. However, the genome sequence of strain R33 shared 76.93–81.47% ANI and had a 19.60–52.40% *in silico* DNA–DNA hybridization (*is*DDH) score with the species-classified *Providencia* strains (Supplementary Table S3). They were too low to reach the cutoff (≥95–96% for ANI and 70% for *is*DDH) to define a classified bacterial species, which suggested that strain R33 is a new species of the genus *Providencia* (Goris et al., 2007; Hu et al., 2019). According to the criteria for species names (International Code of Nomenclature of Prokaryotes, 2019), we named it *Providencia wenzhouensis* R33. This isolate was deposited in China Center for Type Culture Collection, Wuhan, China (CCTCC AB 2021339).

*Providencia wenzhouensis* (wen.zhou.en’sis. N.L. fem. adj. *wenzhouensis* pertaining to Wenzhou, Zhejiang Province, China, where the type strain was isolated) cells are Gram-negative, non-motile, non-spore-forming, non-gas-producing, rod-shaped. Colonies are creamy white, circular, moist, convex, with entire edges on nutrient agar and translucent on MacConkey agar. The strain is catalase-positive and oxidase-negative, gives positive in methyl-red reaction, citrate utilization, and nitrate reduction. The strain is negative for Voges-Proskauer, indole, urease, and the production of H_2_S. Acid is produced from adonitol, salicin, galactose, arabinose, gluconate, glucose, mannitol, D-mannitol, phenylalanine deaminase, but not from arginine, glycerol, inulin, inositol, lactose, lysine, ornithine, trehalose, maltose, pyruvate, sorbitol, sorbose, sucrose, and urea. Differential biochemical characteristics from other members of the genus *Providencia* include acid production from citrate, mannitol, and salicin but not from urea.

The MICs of 26 antibiotics were tested for strain R33 as shown in Table 2. This strain showed resistance to five antibiotics, including fosfomycin, imipenem, nalidixic acid, and polymyxins B and E, and higher MIC values for ribostamycin (128 μg/ml) and ceftiofur sodium (512 μg/ml, an antibiotic for animals only), which have no established breakpoints (Table 2).

**TABLE 2:** MICs of various antibiotics for five bacterial strains (μg/mL).

**Class**	**Antibiotic**	***P. wenzhouensis* R33**	***E. coli***			
			**pUCP20-*aac(2′)-If*/DH5α**	**pUCP20/DH5α**	**DH5α**	**ATCC 25922**
Aminoglycosides	Amikacin	1	1	1	1	2
	Apramycin	4	4	4	4	8
	Kanamycin	0.25	0.25	0.25	0.25	1
	Gentamicin	1	1	0.25	0.25	0.25
	Micronomicin	1	4	0.5	0.5	1
	Netilmicin	0.5	2	0.25	0.25	0.5
	Neomycin	4	32	0.25	0.25	1
	Paromomycin	16	512	2	2	4
	Ribostamycin	128	512	2	2	4
	Sisomicin	0.5	1	0.25	0.25	0.5
	Streptomycin	4	2	2	2	4
	Tobramycin	0.25	4	0.25	0.25	0.5
Aminocyclitols	Spectinomycin	16	8	8	8	8
β-Lactams	Cefoxitin	8	/	/	/	8
	Ceftiofur sodium	512	/	/	/	/
	Imipenem	8	/	/	/	0.5
	Meropenem	0.5	/	/	/	0.06
Amphenicols	Chloramphenicol	8	/	/	/	1
	Florfenicol	8	/	/	/	2
Quinolones	Ciprofloxacin	0.25	/	/	/	0.0075
	Levofloxacin	1	/	/	/	0.06
	Nalidixic acid	256	/	/	/	2
	Norfloxacin	4	/	/	/	0.03
Polymyxins	Polymyxin B	>256	/	/	/	0.25
	Polymyxin E	>256	/	/	/	0.25
Phosphonic acid derivatives	Fosfomycin	256	/	/	/	2

**FIGURE 1:**
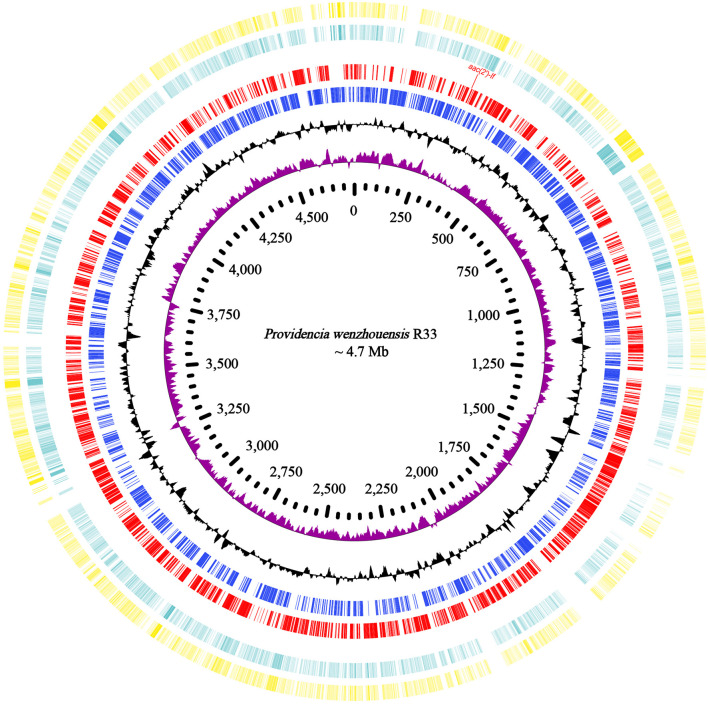
Genome map and comparison of the chromosome sequence of *P. wenzhouensis* R33 with other similar genomes with high identities. From outside to inside: circles 1 and 2 are homologous regions of the chromosomes of *Providencia* sp. 1709051003 (NZ_CP042861.1) and *P. rettgeri* Pr-15-2-50 (NZ_CP039844.1) with *P. wenzhouensis* R33, while the unmatched regions are left blank; circles 3 and 4 display predicted ORFs encoded in the forward and reverse strands, and circles 5, 6, and 7 represent the GC content, GC skew, and scale in kb of the *P. wenzhouensis* R33 chromosome, respectively.

The whole genome of *P. wenzhouensis* R33 was found to consist of a chromosome of approximately 4.75 Mb in length, encoding 4,335 ORFs with an average GC content of 41.57% (Table 3 and Figure 1), and a circular plasmid designated pR33-1, which is 124,159 bp in length and encodes 136 ORFs (Table 3). According to genome sequencing, only three chromosomal genes with a similarity of ≥80% with housekeeping genes, namely, *crp* (encoding cAMP-receptor protein), *rsmA* (encoding translational regulator CsrA), and *E. coli gyrA* (encoding DNA gyrase subunit A) (with similarities of 97.6%, 86.89%, and 83.72%, respectively), were identified. No predicted aminoglycoside resistance genes with similarity ≥80% were found. Among all 11 predicted aminoglycoside resistance-related genes (with similarity <80%) annotated from the whole genome, only one predicted AAC gene [an *aac(2′)-I* homologous gene designated *aac(2′)-If* in this work] had an amino acid sequence similarity of 70.79% with the *aac(2′)-Ia* gene of known function, while the remaining 10 genes (CpxA, KpnH, BaeR, KpnF, KdpE, KpnG, TolC, BaeS, CpxR, and ykkD) encoded for efflux-related proteins (Supplementary Table S4). We subsequently cloned the putative AAC(2′)-I gene (*aac(2′)-If*), and its function was further verified.

**TABLE 3:** General features of the *P. wenzhouensis* R33 genome.

	**Chromosome**	**pR33-1**
Size	4748309	124159
GC content (%)	41.57	41.50
Predicted coding sequences (CDSs) of known proteins	4335	136
Known proteins	3738 (86.23%)	89 (65.44%)
Hypothetical proteins	597 (13.77%)	47 (34.56%)
Protein coding (%)	85.18	81.71
Average ORF length (bp)	923	479
Average protein length (aa)	311	248.7
tRNAs	79	0
rRNA operons	(16S-23S-5S) *6	0
	(16S-23S-5S-5S) *1	

### Homologs of the New Aminoglycoside 2′*-N-*Acetyltransferase

The novel 2′*-N-*acetyltransferase gene *aac(2′)-If* was revealed to be 537 bp in length and to encode a protein of 178 amino acids (ca. 20.33 kDa) with a pI value of 4.47. All 16 of the *aac(2′)-If* homologs (≥65% nucleotide sequence similarity) retrieved from the NCBI nucleotide database were derived from *Providencia* species (mainly *P. rettgeri* and *P. stuartii*). As mentioned above, the predicted protein sequence (no nucleotide sequence available) shared the highest amino acid sequence identity of 95.51% with AAC(2′)-If, which was a hypothetical GNAT family *N*-acetyltransferase (WP_206082813.1) from *P. stuartii*.

**FIGURE 2:**
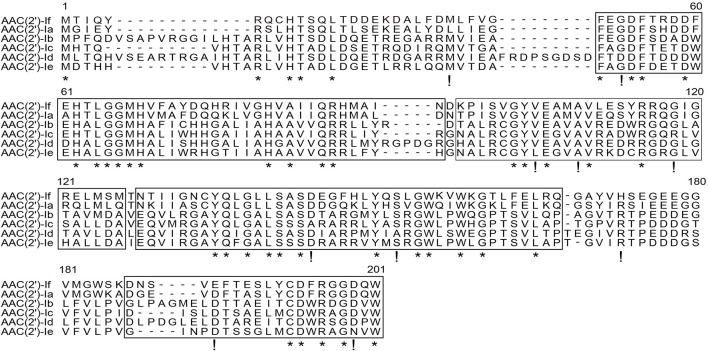
Multiple sequence alignment of the amino acid sequences of the AAC(2′)-I subgroup. The sequences and their accession numbers are as follows: AAC(2′)-If (MW984427), AAC(2′)-Ia (AAA03550.1), AAC(2′)-Ib (AAC44793.1), AAC(2′)-Ic (AAB17563.1), AAC(2′)-Id (AAB41701.1), and AAC(2′)-Ie (CAR72650.1). The numbers on the right correspond to the amino acid residues in each full-length protein, with the conserved motif sites boxed, fully conserved residues shown with asterisks, and highly similar residues shown with exclamation marks.

**FIGURE 3:**
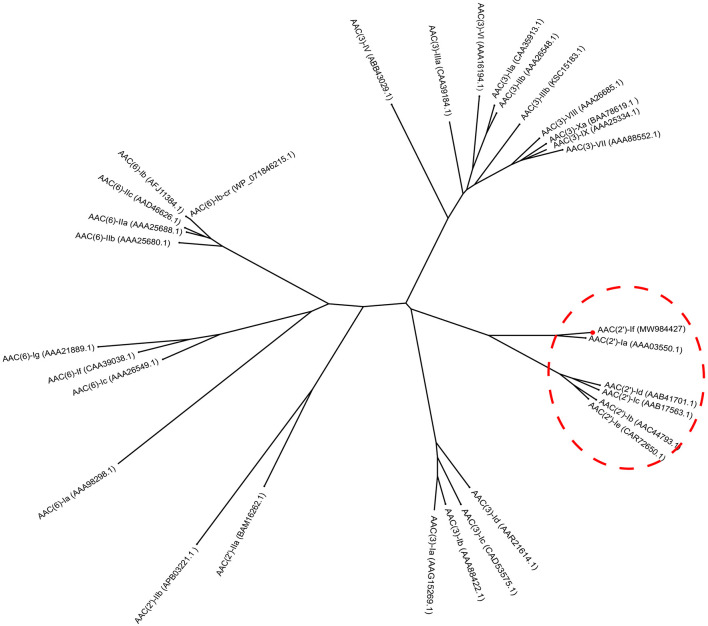
Phylogenetic tree showing the relationship of AAC(2′)-If with other chromosome-borne acetyltransferases. AAC(2′)-If from this study is shown with a red dot.

The results of multiple sequence alignment of AAC(2′) enzymes showed that AAC(2′)-If shared at most 70.79%, 35.03%, 35.26%, 30.10%, and 31.21% identity with the five AAC(2′)-I subgroups AAC(2′)-Ia (AAA03550.1), AAC(2′)-Ib (AAC44793.1), AAC(2′)-Ic (AAB17563.1), AAC(2′)-Id (AAB41701.1), and AAC(2′)-Ie (CAR72650.1), respectively. Except for the AAC(2′)-Ia subgroup, which had the highest identity value of more than 70.0%, the other four subgroups showed less than 40.0% identity (Figure 2). A phylogenetic tree showed that AAC(2′)-If clustered closest to AAC(2′)-Ia (Figure 3).

### Functional Characteristics of the *aac(2′)-If* Gene

To investigate the functional role of AAC(2′)-If, the sequence encoding the ORF of AAC(2′)-If with its promoter region was cloned into the pUCP20 vector, and the recombinant plasmid (pUCP20-*aac(2′)-If*) was transformed into *E. coli* DH5α. The MICs of several aminoglycoside antibiotics against the transformant harboring *aac(2′)-If* (pUCP20-*aac(2′)-If*/DH5α) were determined. The presence of plasmid borne *aac(2′)-If* in *E. coli* led to elevated MICs of ribostamycin, neomycin, paromomycin, tobramycin, micronomicin, netilmicin, gentamicin, and sisomicin (256-, 128-, 256-, 16-, 8-, 8-, 4-, and 4-fold increases, respectively) in comparison with those for the control strains (DH5α or DH5α carrying the vector pUCP20) (Table 2). However, as expected, no changes in MICs of streptomycin and spectinomycin were observed. Additionally, the susceptibility of the *aac(2′)-If*-carrying R33 to aminoglycosides and other antibiotics are also shown in Table 2, with relatively high MIC values (≥4 μg/mL) observed for several aminoglycosides (i.e., apramycin, neomycin, paromomycin, ribostamycin, and streptomycin), spectinomycin, polymyxins B and E, nalidixic acid, norfloxacin, chloramphenicol, florfenicol, cefoxitin, ceftiofur, imipenem, and fosfomycin.

### Kinetic Parameters of AAC(2′)-If

Investigation of the acetyltransferase activity and kinetic parameters of AAC(2′)-If showed that of the 10 aminoglycosides tested, the enzyme was able to acetylate ribostamycin, neomycin, tobramycin, micronomicin, sisomicin, and gentamicin, but worse acetyltransferase activity was detected for netilmicin and kanamycin (Table 4). The highest catalytic efficiency was observed with ribostamycin [*k_*cat*_/K_*m*_* ratio was (3.72 ± 0.52) × 10^4^ M^–1^^⋅^s^–1^], which was found to be the best aminoglycoside substrate, whereas kanamycin was the worst substrate [*k_*cat*_/K_*m*_* ratio = (8.71 ± 1.25) × 10^3^ M^–1^^⋅^s^–1^]. These differences appeared to result from large differences in the turnover rates (*k*_*cat*_), which varied by almost six-fold compared to the affinity (Table 4).

**TABLE 4:** Steady-state kinetic parameters for AAC(2′)-If.

**Substrate**	***K*_*cat*_ (s^–1^)**	***K*_*m*_ (μM)*^a^***	***K_*cat*_/K_*m*_* (M^–1^^⋅^s^–1^)**
Gentamicin	0.273 ± 0.006	19.92 ± 2.9	(1.37 ± 0.16) × 10^4^
Kanamycin	0.021 ± 0.018	2.37 ± 0.29	(8.71 ± 1.25) × 10^3^
Micronomicin	0.323 ± 0.033	21.42 ± 3.9	(1.51 ± 0.15) × 10^4^
Neomycin	0.061 ± 0.002	3.06 ± 0.55	(2.01 ± 0.39) × 10^4^
Netilmicin	0.44 ± 0.015	41.11 ± 4.36	(1.07 ± 0.07) × 10^4^
Paromomycin	0.113 ± 0.007	6.88 ± 1.43	(1.65 × 0.29) × 10^4^
Ribostamycin	0.164 ± 0.01	4.41 ± 0.86	(3.72 ± 0.52) × 10^4^
Sisomicin	0.203 ± 0.007	7.03 ± 0.94	(2.88 ± 0.49) × 10^4^
Tobramycin	0.124 ± 0.021	15.24 ± 4.6	(0.82 ± 0.1) × 10^4^
Streptomycin	NA	NA	NA

*^*a*^ Values are means ± standard deviations.*

*A, no acyl transfer activity was detected.*

### *aac(2′)-If* RNA Expression After Exposure to Antibiotics

**FIGURE 4:**
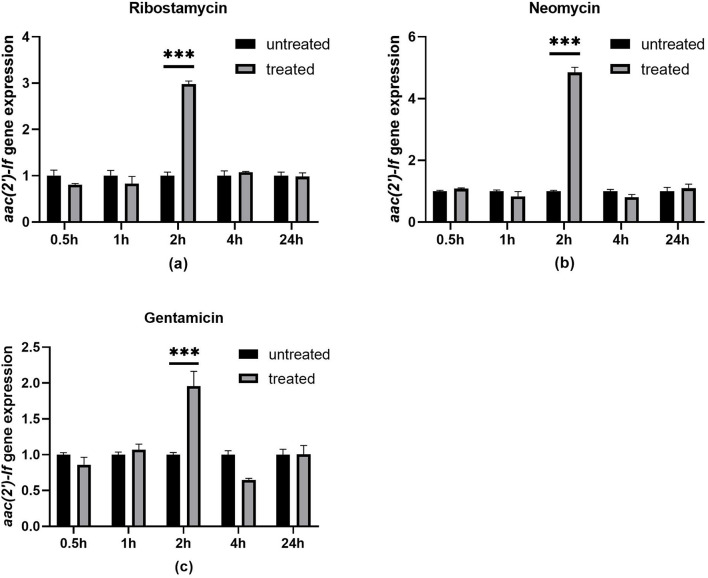
Expression levels of *aac(2′)-If* treated or untreated with 32 μg/mL ribostamycin **(a)**, 1 μg/mL neomycin **(b)**, and 0.25 μg/mL gentamicin **(c)**. Bars represent means ± standard errors and experiments were performed in triplicate. ^∗∗∗^Presents significant difference, *P* < 0.001.

To investigate the effect of aminoglycoside antibiotics on the *aac(2′)-If* gene expression, we determined the change of the *aac(2′)-If* gene expression levels with or without ribostamycin, neomycin, and gentamicin for different periods of time. Quantitative real-time PCR indicated that the expression of the *aac(2′)-If* gene increased significantly when the cells were induced with the three aminoglycoside antibiotics for 2 h (*P* < 0.001). The expression level increased approximately three-, five-, and two-fold when treated with ribostamycin, neomycin, and gentamicin, respectively. No significant difference was observed between any pair of the other groups (Figure 4).

### Genetic Context of the *aac(2′)-If* Gene

A total of 39 sequences (including one from this work) that were approximately 21 kb in length, with *aac(2′)-If-like* genes in the center, that shared nucleotide sequence similarities between 32.18 and 70.79% with *aac(2′)-If* were retrieved from the database. These sequences were all from *Providencia* (68.42%, 26/38) and *Proteus* strains (31.58%, 12/38). The genes from *Providencia* all shared amino acid sequence similarities of ≥54.61% with *aac(2′)-If*, while the genes from *Proteus* showed amino acid similarities of below 40.0% (except one of 68.76% and two of 53.39%) with *aac(2′)-If*.

**FIGURE 5:**
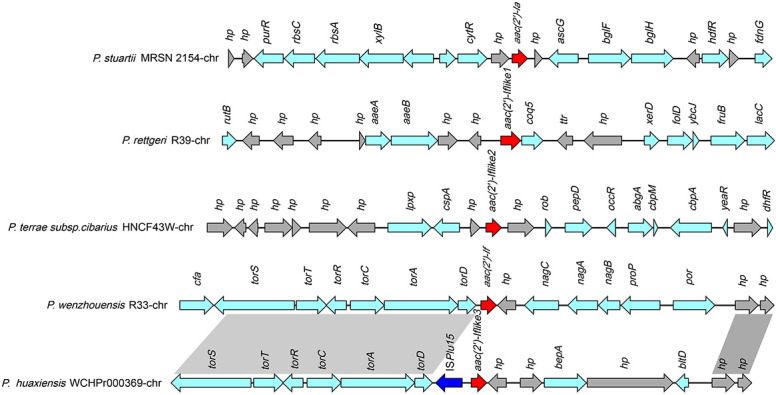
Comparative analysis of the genomic context of the *aac(2′)-If* gene with similar sequences. Homologous genes are shown in the same colors, and the genes with no homologs are colored gray. The accession numbers of the sequences are as follows: *P. stuartii* MRSN 2154 (CP003488.1), *P. rettgeri* R39 chromosome (CP066315.1), *P. huaxiensis* WCHP000369 chromosome (CP031123.2), and *P. terrae* subsp. *cibarius* HNCF44W (CP053042.1).

The 39 sequences could be clustered into five groups according to the sequence similarities. The sequence of strain R33 was grouped alone, while the other four groups contained six or more sequences each (Supplementary Table S5). One sequence that shared the highest similarity with the other sequences in the same group was selected as a representative to perform the sequence structure comparison within the groups (Figure 5). The sequence of *aac(2′)-If* of R33 only showed similar sequence structures upstream of the *aac(2′)-If* gene as that of group 3, and the other sequences or sequence regions from these groups were completely different from each other.

## Discussion

In this work, by correlation analysis of the aminoglycoside resistance phenotypes with the genotypes, we identified a novel resistance gene named as *aac(2′)-If*. Homology analysis of AAC(2′)-If with the sequences in the NCBI nonredundant protein database demonstrated that the sequence that shared the highest amino acid sequence identity with AAC(2′)-If was that of AAC(2′)-Ia, a protein of known function, sharing an amino acid sequence identity of only 70.79%. Moreover, only 35 predicted protein sequences with identities between 60.0% and 95.51% with AAC(2′)-If were found, and most of the sequences were derived from the chromosomes of *Providencia* species (especially *P. rettgeri* and *P. stuartii*). Only one protein described as a GNAT family *N*-acetyltransferase was from *Morganellaceae*, and one was from an unclassified *Shigella* species. This result indicated that to clarify the evolution of the *aac(2′)-If* gene, further studies for genotypes with AACs of higher identities are warranted.

The antibiotic resistance profile conferred by AAC(2′)-If was basically the same as that of other AAC(2′)-I enzymes. They conferred resistance to a variety of aminoglycosides, including 4,5-disubstituted aminoglycosides (ribostamycin, neomycin, paromomycin) and 4,6-disubstituted aminoglycosides (gentamicin, tobramycin, micronomicin, netilmicin, and sisomicin) (Ainsa et al., 1996; Franklin and Clarke, 2001). As expected, in this work, the recombinant strain carrying *aac(2′)-If* remained susceptible to spectinomycin and streptomycin, which does not contain 2′ amino groups in their chemical structure. Although AAC(2′)-If enzymes can modify both 4,6- and 4,5-disubstituted aminoglycosides, the extent of modification was different. 4,5-Disubstituted aminoglycosides were generally better substrates for AAC(2′)-If. Ribostamycin was the most efficient substrate examined and exhibited a 7-fold higher *k*_*cat*_ value than kanamycin, with a 256-fold increase in the MIC value. Additionally, AAC(2′)-If exhibited weak acetylation against kanamycin [*k_*cat*_/K_*m*_* = (8.71 ± 1.25) × 10^3^ M^–1^^⋅^s^–1^] in enzyme kinetic analysis, which may explain why this enzyme is unable to confer resistance to kanamycin *in vivo*. The MIC values of kanamycin for both the recombinant and the control strains remained the same (0.25 μg/mL). AAC(2′)-If appeared to be less active against aminoglycoside substrates than His-AAC(2′)-Ia and AAC(2′)-Ic enzymes by one or two orders of magnitude (Hegde et al., 2001). The *K*_*m*_ value for these three enzymes did not vary greatly, but the turnover number *k*_*cat*_ values differed more than 10-fold between them (Cox et al., 2018).

According to the result of comparative analysis with homologous genes, except for sequences from one group, which was composed of sequences from *Providencia* that shared a similar upstream sequence with *aac(2′)-If*, the sequences from the other groups showed completely different sequence structures compared to that of *aac(2′)-If.* Considering that *aac(2′)-If* belongs to a novel species of the genus *Providencia* and only one genome sequence from this work is available, and considering that this sequence shared only 70.79% sequence similarity with all of the sequences available in the NCBI nucleotide database, the evolutionary study of this novel aminoglycoside 2′*-N-*acetyltransferase gene could be performed when a greater number of whole-genome sequences carrying phylogenetically close *aac(2′)-If*-like genes become available in the future.

## Conclusion

In the current study, we identified *aac(2′)-If* encoded on the chromosome of an isolate of a new *Providencia* species, designated *P. wenzhouensis* R33, with the complete genome sequenced. The novel aminoglycoside 2′*-N-*acetyltransferase AAC(2′)-If shares the highest amino acid sequence identity of 70.79% with the functionally characterized AAC(2′)-Ia and confers stronger resistance to ribostamycin and neomycin. Whole-genome sequencing revealed that this novel resistance gene was encoded in the chromosome and was not related to any MGEs. Identification of a novel resistance gene in a novel bacterial species will help us find ways to explore the increasing number of resistance mechanisms in the microbial population and to further elucidate the intrinsic resistance mechanisms of unusual microorganisms.

## Data Availability Statement

The datasets presented in this study can be found in online repositories. The names of the repository/repositories and accession number(s) can be found in the article/Supplementary Material.

## Ethics Statement

This study uses strains obtained from an anal swab of a rabbit on an animal farm in Wenzhou, China. It did not require the study to be reviewed or approved by an ethics committee because the animal was not presented in this study. The animal legal guardian provided written informed consent to participate in this study.

## Author Contributions

HZ, MZ, and QB contributed to the conception and design of study. JLL, XL, WS, and MG contributed to the acquisition of data. KZ, JLL, CF, and KL contributed to the data analysis and interpretation. KZ, JWL, XD, PZ, and QB contributed to the drafting of manuscript. KZ, JLL, WS, MG, QL, and XZ performed the experiments. All authors contributed to the article and approved the submitted version.

## Conflict of Interest

The authors declare that the research was conducted in the absence of any commercial or financial relationships that could be construed as a potential conflict of interest.

## Publisher’s Note

All claims expressed in this article are solely those of the authors and do not necessarily represent those of their affiliated organizations, or those of the publisher, the editors and the reviewers. Any product that may be evaluated in this article, or claim that may be made by its manufacturer, is not guaranteed or endorsed by the publisher.

## Footnotes

1
https://lpsn.dsmz.de/search?word=Providencia

2
http://www.softberry.com/berry.phtml?topic=bprom&group=programs&subgroup=gfindb

3
https://web.expasy.org/protparam/

4
https://www.python.org/
