# Supplementary material for: Identification and Characterization of a Novel Chromosomal Aminoglycoside 2′-N-Acetyltransferase, AAC(2′)-If, From an Isolate of a Novel Providencia Species, Providencia wenzhouensis R33
Source: Front Microbiol. 2021 Nov 19;12:711037. doi: 10.3389/fmicb.2021.711037 (PMC8640171; doi:10.3389/fmicb.2021.711037)
Supplement: Supplementary file 1 [file Table_1.DOCX]

**TABLE S1 | Cloning primers for the *aac(2’)-If* gene**

| Primer^a^ | Sequence (5’–3’)^b^ | Restriction endonuclease | Vector | Annealing  temperature (◦C) | Amplicon size (bp) |
| --- | --- | --- | --- | --- | --- |
| pro-*aac(2’)-If*-F | CGGGATCCGATGCCCTTACGCATCGACC | *Bam*HⅠ | pUCP20 | 56 | 886 |
| pro- *aac(2’)-If* -R | CCAAGCTTTTACCATTGATCGCCACCGCG | *Hin*dⅢ | pUCP20 |  |  |
| orf-*aac(2’)-If*-F | CGCGGATCCCTGGTGCCGCGCGGCAGCATGACTATTCAATACAGGCAATGCCACACTTC | *Bam*HⅠ+Thrombin | pCold I | 58 | 570 |
| orf-*aac(2’)-If*-R | CCCAAGCTTTTACCACTGATCGCCACCGCGAAAGTCG | *Hin*dⅢ | pCold I |  |  |

^a^ primers with “orf” were used to clone the ORF of the *aac(2’)-If* gene, and primers with “pro” were used to clone the *aac(2’)-If* gene with its promoter region.

^b^ The underlined sequences represent the restriction endonuclease sites and their protective bases.
