# Supplementary material for: Identification and Characterization of a Novel Chromosomal Aminoglycoside 2′-N-Acetyltransferase, AAC(2′)-If, From an Isolate of a Novel Providencia Species, Providencia wenzhouensis R33
Source: Front Microbiol. 2021 Nov 19;12:711037. doi: 10.3389/fmicb.2021.711037 (PMC8640171; doi:10.3389/fmicb.2021.711037)
Supplement: Supplementary file 2 [file Table_2.docx]

**TABLE S2 | Primers for Q-PCR**

| Primer^a^ | Sequence (5’–3’) | Annealing temperature (◦C) | Amplicon size (bp) |
| --- | --- | --- | --- |
| q-*aac(2’)-If*-F | CAATACAGGCAATGCCACAC | 60 | 249 |
| q-*aac(2’)-If*-R | ACTTTCCAGTACCGCCATTG |  |  |
| R33-16S-F | ACAGAAGAAGCACCGGCTAA | 60 | 208 |
| R33-16S-R | CATTTCACCGCTACACATGG |  |  |

^a^ primers with “q” were used for the *aac(2’)-If* gene, and primers with “16S” was used for the 16S RNA gene of R33.
