# Supplementary material for: Identification and Characterization of a Novel Chromosomal Aminoglycoside 2′-N-Acetyltransferase, AAC(2′)-If, From an Isolate of a Novel Providencia Species, Providencia wenzhouensis R33
Source: Front Microbiol. 2021 Nov 19;12:711037. doi: 10.3389/fmicb.2021.711037 (PMC8640171; doi:10.3389/fmicb.2021.711037)
Supplement: Supplementary file 3 [file Table_3.DOCX]

**TABLE S3 | ANI and *is*DDH scores between strain R33 and the type strains of *Providencia* species**

| Species | Strain | Accession | ANI (%) | *is*DDH |
| --- | --- | --- | --- | --- |
| *Providencia alcalifaciens* | FDAARGOS_408 | NZ_CP023536.1 | 77.97 | 21.90 |
| *Providencia rettgeri* | AR_0082 | NZ_CP029736.1 | 81.47 | 52.40 |
| *Providencia stuartii* | ATCC 33672 | NZ_CP008920.1 | 77.14 | 21.40 |
| *Providencia rustigianii* | NCTC6933 | NZ_LR134189.1 | 77.93 | 27.20 |
| *Providencia huaxiensis* | WCHPr000369 | NZ_CP031123.2 | 81.36 | 47.90 |
| *Providencia vermicola* | P8538 | NZ_CP048796.1 | 77.18 | 21.50 |
| *Providencia sneebia* | DSM 19967 | NZ_CM001773.1 | 76.93 | 21.20 |
| *Providencia heimbachae* | NCTC12003 | NZ_LS483422.1 | 79.17 | 22.30 |
| *Providencia burhodogranariea* | DSM 19968 | NZ_KB233222.1 | 77.02 | 19.60 |
| *Providencia thailandensis* | KCTC 23281 | NZ_BMYH01000001.1 | 77.17 | 22.30 |
