# Supplementary material for: Identification and Characterization of a Novel Chromosomal Aminoglycoside 2′-N-Acetyltransferase, AAC(2′)-If, From an Isolate of a Novel Providencia Species, Providencia wenzhouensis R33
Source: Front Microbiol. 2021 Nov 19;12:711037. doi: 10.3389/fmicb.2021.711037 (PMC8640171; doi:10.3389/fmicb.2021.711037)
Supplement: Supplementary file 4 [file Table_4.DOCX]

**TABLE S4 | Aminoglycoside resistance-associated putative resistance genes identified in *P. wenzhoubz* R33**

| Location | Putative resistance proteins | Similarity | Antibiotic resistance pattern ^a^ | Resistance mechanism |
| --- | --- | --- | --- | --- |
| Chromosome | CpxA | 77.41% | aminocoumarin antibiotic; aminoglycoside antibiotic | antibiotic efflux |
| Chromosome | KpnH | 75.54% | penem; cephalosporin; macrolide antibiotic; carbapenem; aminoglycoside antibiotic; peptide antibiotic; penam; fluoroquinolone antibiotic | antibiotic efflux |
| Chromosome | AAC(2')-Ia | 70.79% | aminoglycoside antibiotic | antibiotic inactivation |
| Chromosome | BaeR | 69.69% | aminocoumarin antibiotic; aminoglycoside antibiotic | antibiotic efflux |
| Chromosome | KpnF | 68.17% | aminoglycoside antibiotic; rifamycin antibiotic; macrolide antibiotic; peptide antibiotic; cephalosporin; tetracycline antibiotic | antibiotic efflux |
| Chromosome | KdpE | 66.96% | aminoglycoside antibiotic | antibiotic efflux |
| Chromosome | KpnG | 65.14% | penem; cephalosporin; macrolide antibiotic; carbapenem; aminoglycoside antibiotic; peptide antibiotic; penam; fluoroquinolone antibiotic | antibiotic efflux |
| Chromosome | TolC | 60.44% | penem; tetracycline antibiotic; aminocoumarin antibiotic; cephalosporin; macrolide antibiotic; carbapenem; aminoglycoside antibiotic; glycylcycline; rifamycin antibiotic; peptide antibiotic; penam; triclosan; cephamycin; fluoroquinolone antibiotic; phenicol antibiotic | antibiotic efflux |
| Chromosome | BaeS | 53.68% | aminocoumarin antibiotic; aminoglycoside antibiotic | antibiotic efflux |
| Chromosome | CpxR | 44.83% | sulfonamide antibiotic; penem; tetracycline antibiotic; aminocoumarin antibiotic; cephalosporin; macrolide antibiotic; carbapenem; cephamycin; peptide antibiotic; diaminopyrimidine antibiotic; penam; aminoglycoside antibiotic; monobactam; fluoroquinolone antibiotic; phenicol antibiotic | antibiotic efflux |
| Chromosome | ykkD | 39.42% | tetracycline antibiotic; aminoglycoside antibiotic; phenicol antibiotic | antibiotic efflux |

^­­a^ Antibiotic resistance pattern of putative resistance genes.
