# Supplementary material for: Identification and Characterization of a Novel Chromosomal Aminoglycoside 2′-N-Acetyltransferase, AAC(2′)-If, From an Isolate of a Novel Providencia Species, Providencia wenzhouensis R33
Source: Front Microbiol. 2021 Nov 19;12:711037. doi: 10.3389/fmicb.2021.711037 (PMC8640171; doi:10.3389/fmicb.2021.711037)
Supplement: Supplementary file 5 [file Table_5.DOCX]

**TABLE S5 | Details of 38 *aac(2')-If* homologous genes**

| Accession number | Source | Description | Coverage^a^ | Identity^a^ | Similarity^a^ | Group |
| --- | --- | --- | --- | --- | --- | --- |
| CP066315.1 | *Providencia rettgeri* R39 | chromosome, complete genome | 100 | 70.79 | 70.79 | 1 |
| CP059345.1 | *Providencia rettgeri* 2055 | chromosome, complete genome | 100 | 70.79 | 70.79 | 1 |
| CP027418.1 | *Providencia rettgeri* FDAARGOS_330 | chromosome, complete genome | 100 | 70.61 | 70.61 | 1 |
| CP017671.1 | *Providencia rettgeri* RB151 | complete genome | 100 | 70.61 | 70.61 | 1 |
| CP054158.1 | *Providencia rettgeri* HH18 | chromosome, complete genome | 100 | 70.24 | 70.24 | 1 |
| CP034667.1 | *Proteus vulgaris* PvSC3 | plasmid pPvSC3, complete sequence | 100 | 68.76 | 68.76 | 1 |
| MT813046.1 | *Providencia stuartii* M2 | plasmid pM2-1, complete sequence | 100 | 68.76 | 68.76 | 1 |
| AP022374.1 | *Providencia stuartii* BML2537 | DNA, complete genome | 91 | 71.49 | 65.06 | 2 |
| CP044076.1 | *Providencia stuartii* FDAARGOS_645 | chromosome, complete genome | 91 | 71.49 | 65.06 | 2 |
| CP026704.1 | *Providencia stuartii* AR_0026 | complete genome | 91 | 71.49 | 65.06 | 2 |
| CP014024.2 | *Providencia stuartii* FDAARGOS_145 | complete genome | 91 | 71.49 | 65.06 | 2 |
| CP008920.1 | *Providencia stuartii* ATCC 33672 | complete genome | 91 | 71.49 | 65.06 | 2 |
| CP066071.1 | *Providencia stuartii* FDAARGOS_1040 | chromosome, complete genome | 91 | 71.49 | 65.06 | 2 |
| CP065420.1 | *Providencia* sp. *2.29* | chromosome, complete genome | 91 | 71.49 | 65.06 | 2 |
| CP048621.1 | *Providencia stuartii* MF1 | chromosome, complete genome | 91 | 71.49 | 65.06 | 2 |
| CP031508.1 | *Providencia stuartii* FDAARGOS_87 | chromosome, complete genome | 91 | 71.29 | 64.87 | 2 |
| CP027398.1 | *Providencia stuartii* FDAARGOS_291 | chromosome, complete genome | 91 | 71.29 | 64.87 | 2 |
| CP017054.1 | *Providencia stuartii* BE2467 | complete genome | 91 | 71.29 | 64.87 | 2 |
| CP003488.1 | *Providencia stuartii* MRSN 2154 | complete genome | 91 | 71.29 | 64.87 | 2 |
| CP048796.1 | *Providencia vermicola* P8538 | chromosome, complete genome | 84 | 70.56 | 59.27 | 2 |
| CP067099.1 | *Providencia vermicola* LLDRA6 | chromosome, complete genome | 80 | 68.26 | 54.61 | 2 |
| CP031123.2 | *Providencia huaxiensis* WCHPr000369 | chromosome, complete genome | 82 | 71.78 | 58.86 | 3 |
| CP062821.1 | *Providencia rettgeri* Res13-Sevr-LER2-35 | chromosome, complete genome | 82 | 71.78 | 58.86 | 3 |
| CP059347.1 | *Providencia rettgeri* 2353217 | Chromosome, complete genome | 82 | 71.78 | 58.86 | 3 |
| CP042860.1 | *Providencia* sp. 1701091 | chromosome, complete genome | 82 | 71.24 | 58.42 | 3 |
| CP042859.1 | *Providencia* sp. 1701011 | chromosome, complete genome | 82 | 71.24 | 58.42 | 3 |
| CP029736.1 | *Providencia rettgeri* AR_0082 | chromosome, complete genome | 82 | 71.24 | 58.42 | 3 |
| CP032663.1 | *Proteus vulgaris* CCU063 | chromosome, complete genome | 80 | 66.74 | 53.39 | 4 |
| CP034105.1 | *Proteus* sp. GOKU | chromosome, complete genome | 80 | 66.74 | 53.39 | 4 |
| CP047639.1 | *Proteus* sp. ZN5 | chromosome, complete genome | 58 | 68.34 | 39.64 | 4 |
| CP053042.1 | *Proteus terrae* subsp. *cibarius* HNCF44W | chromosome, complete genome | 46 | 70.36 | 32.37 | 4 |
| CP053044.1 | *Proteus terrae* subsp. *cibarius* HNCF43W | chromosome, complete genome | 46 | 70.36 | 32.37 | 4 |
| CP053371.1 | *Proteus terrae* subsp. *cibarius* G32 | chromosome, complete genome | 46 | 70.36 | 32.37 | 4 |
| CP047286.1 | *Proteus terrae* subsp. *cibarius* G11 | chromosome, complete genome | 46 | 70.36 | 32.37 | 4 |
| CP047349.1 | *Proteus terrae* subsp. *cibarius* ZN2 | chromosome, complete genome | 46 | 69.96 | 32.18 | 4 |
| CP047340.1 | *Proteus terrae* subsp. *cibarius* ZF1 | chromosome, complete genome | 46 | 69.96 | 32.18 | 4 |
| CP034668.1 | *Proteus vulgaris* PvSC3 | chromosome, complete genome | 46 | 69.96 | 32.18 | 4 |
| CP045008.1 | *Proteus terrae* subsp. *cibarius* ZF2 | chromosome, complete genome | 46 | 69.96 | 32.18 | 4 |

^a^ comparison between *aac(2’)-If-*homologous genes and *aac(2’)-If*
